# Supplementary material for: Diversity and metagenome analysis of a hydrocarbon-degrading bacterial consortium from asphalt lakes located in Wietze, Germany
Source: AMB Express. 2021 Jun 14;11:89. doi: 10.1186/s13568-021-01250-4 (PMC8203775; doi:10.1186/s13568-021-01250-4)
Supplement: Supplementary file 1 — Additional file 1: Figure S1. Bacterial taxonomic distribution of all samples based on 16S rRNA gene amplicon data. Table S1. Richness, diversity and evenness obtained from the 16S rRNA sequencing of sampling sites and enrichment cultures. Table S3. Quality check for the MAGs. [file 13568_2021_1250_MOESM1_ESM.pdf]

Diversity and metagenome analysis of a hydrocarbon-degrading bacterial consortium from asphalt lakes located in Wietze, Germany

Michael O. Eze[1,2*], Grant C. Hose[3], Simon C. George[2] and Rolf Daniel[1]

[1]Department of Genomic and Applied Microbiology and Göttingen Genomics Laboratory, Georg-August University of Göttingen, 37077 Göttingen, Germany.

[2]Department of Earth and Environmental Sciences, Macquarie University, Sydney, NSW 2109, Australia.

[3]Department of Biological Sciences, Macquarie University, Sydney, NSW 2109, Australia.

***Correspondence:** meze@gwdg.de

Supplementary Materials

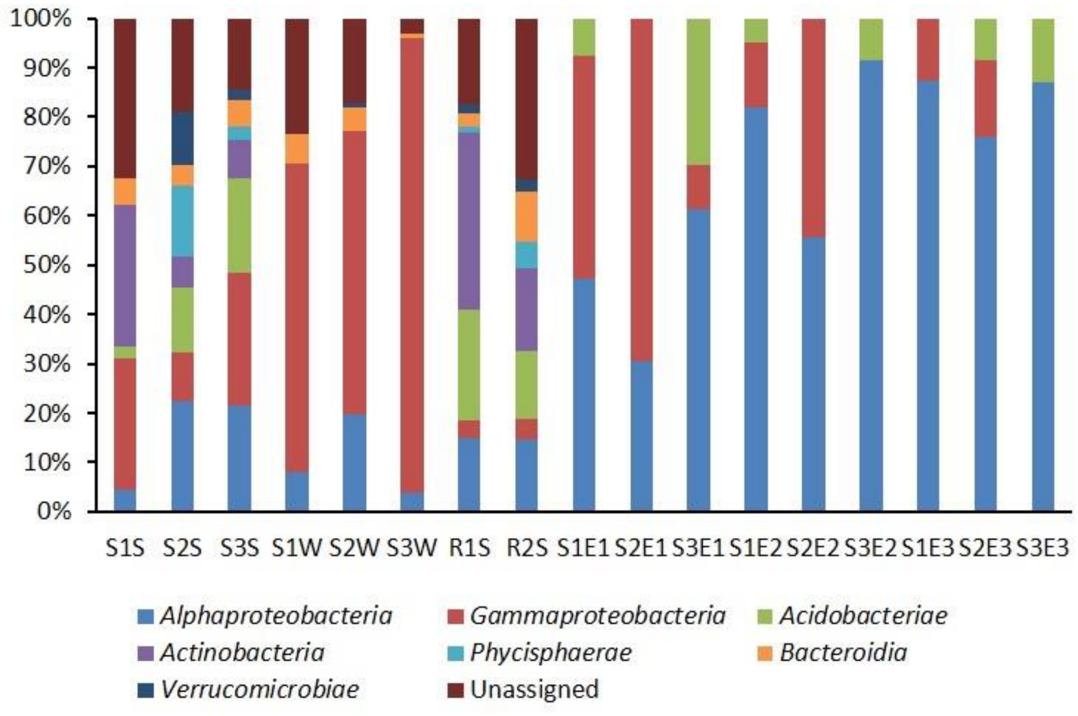

Fig. S1. Bacterial taxonomic distribution of all samples based on 16S rRNA gene amplicon data.

Table S1. Richness, diversity and evenness obtained from the 16S rRNA sequencing of sampling sites and enrichment cultures

Sample ID	Name	Richness	Chao1	Chao1 (%)	Shannon Diversity	Pielou's Evenness
X20200114.BEWE.a.2136_S261	S1S	381.99	475.51	80.33	4.140482857	0.696418531
X20200114.BEWE.a.2137_S262	S2S	958.43	1166.75	82.15	5.927652696	0.863422675
X20200114.BEWE.a.2138_S263	S3S	885.54	1076.78	82.24	5.625668073	0.828986773
X20200114.BEWE.a.2139_S264	S1W	731.44	971.95	75.25	4.491982504	0.68111784
X20200114.BEWE.a.2140_S265	S2W	666.94	848.21	78.63	3.840835511	0.590652415
X20200114.BEWE.a.2141_S266	S3W	389	581.5	66.9	2.409478261	0.40403223
X20200114.BEWE.a.2142_S267	R1S	629.32	806.44	78.04	4.89315041	0.759258936
X20200114.BEWE.a.2143_S268	R2S	1168.08	1274.35	91.66	6.322569919	0.89515298
X20200114.BEWE.a.2144_S269	S1E1	62.34	67.76	92	2.684138231	0.649503002
X20200114.BEWE.a.2145_S270	S2E1	44.91	50.35	89.2	2.228003222	0.585598434
X20200114.BEWE.a.2146_S271	S3E1	38.41	40.95	93.8	1.755745383	0.481247923
X20200114.BEWE.a.2147_S272	S1E2	45.09	56.52	79.78	1.896011697	0.497815886
X20200114.BEWE.a.2148_S273	S2E2	31.88	35.16	90.68	1.921605494	0.555059859
X20200114.BEWE.a.2149_S274	S3E2	14.05	14.66	95.82	0.829529079	0.313903749
X20200114.BEWE.a.2150_S275	S1E3	23.26	30.09	77.3	1.761694985	0.55984851
X20200114.BEWE.a.2151_S276	S2E3	27.44	40.95	67	1.257543211	0.379692792
X20200114.BEWE.a.2152_S277	S3E3	15.28	19.03	80.3	0.962736927	0.353097786

Table S3. Quality check for the MAGs

Bin.Id	Marker.lineage	X..genomes	X..markers	X..marker.sets	X0	X1	X2	X3	X4	X5.	Completeness	Contamination	Strain. Hetero-geneity
BEWE_m_45_metabat2.1	k__Bacteria (UID3187)	2258	187	116	54	125	8	0	0	0	82.75	6.47	12.5
BEWE_m_45_metabat2.11	o__Actinomycetales (UID1814)	148	572	276	25	547	0	0	0	0	97.53	0	0
BEWE_m_45_metabat2.15	o__Rhodospirillales (UID3754)	63	336	201	48	284	4	0	0	0	93.75	1.49	25
BEWE_m_45_metabat2.18	c__Gammaproteobacteria (UID4202)	67	481	276	110	329	41	0	1	0	77.26	8.06	0
BEWE_m_45_metabat2.19	k__Bacteria (UID3187)	2258	188	117	55	132	1	0	0	0	87.39	0.85	0
BEWE_m_45_metabat2.21	f__Xanthomonadaceae (UID4214)	55	659	290	103	536	20	0	0	0	91.72	3.88	30
BEWE_m_45_metabat2.22	c__Gammaproteobacteria (UID4201)	1164	275	174	93	179	3	0	0	0	64.27	1.72	66.67
BEWE_m_45_metabat2.25	c__Gammaproteobacteria (UID4267)	119	544	284	28	513	3	0	0	0	94.55	0.82	0
BEWE_m_45_metabat2.26	k__Bacteria (UID203)	5449	104	58	48	52	4	0	0	0	66.07	6.03	50
BEWE_m_45_metabat2.27	c__Betaproteobacteria (UID3888)	323	387	234	41	340	5	1	0	0	89.01	0.88	25
BEWE_m_45_metabat2.28	k__Bacteria (UID203)	5449	102	56	13	89	0	0	0	0	80.36	0	0
BEWE_m_45_metabat2.33	f__Actinomycetaceae (UID1531)	42	420	211	58	358	2	2	0	0	87.1	2.37	25
BEWE_m_45_metabat2.35	c__Gammaproteobacteria (UID4267)	119	544	284	44	493	7	0	0	0	90.77	1.88	14.29
BEWE_m_45_metabat2.41	o__Rhodospirillales (UID3754)	63	336	201	114	219	3	0	0	0	65.49	0.76	66.67
BEWE_m_45_metabat2.6	g__Burkholderia (UID4006)	64	769	248	51	692	26	0	0	0	94.03	1.51	38.46
BEWE_m_46_metabat2.2	o__Rhodospirillales (UID3754)	63	336	201	80	256	0	0	0	0	72.39	0	0
BEWE_m_46_metabat2.4	k__Bacteria (UID3187)	2258	188	117	1	186	1	0	0	0	99.79	0.85	0
BEWE_m_46_metabat2.5	o__Rhodospirillales (UID3754)	63	336	201	0	335	1	0	0	0	100	0.08	0
